# Flavobacterium facile sp. nov., isolated from water system of Atlantic salmon (Salmo salar) fry cultured in Chile

**DOI:** 10.1099/ijsem.0.006468

**Published:** 2024-07-26

**Authors:** Rute Irgang, Mónica Saldarriaga-Córdoba, Matías Poblete-Morales, Ruben Avendaño-Herrera

**Affiliations:** 1 Universidad Andrés Bello, Laboratorio de Patología de Organismos Acuáticos y Biotecnología Acuícola, Facultad de Ciencias de la Vida, Viña del Mar, Chile; 2 Interdisciplinary Center for Aquaculture Research (INCAR), Viña del Mar, Chile; 3 Escuela de Medicina Veterinaria & Centro de Investigación en Recursos Naturales y Sustentabilidad (CIRENYS), Universidad Bernardo O´Higgins, Santiago, Chile; 4 Centro de Investigación Marina Quintay (CIMARQ), Universidad Andrés Bello, Quintay, Valparaíso, Chile

**Keywords:** Atlantic salmon, Chile, fry, *Flavobacterium facile*

## Abstract

Strain T-12^T^, an orange, Gram-stain-negative, non-motile, rod-shaped strain, was isolated in November 2013 from water samples collected from an Atlantic salmon (*Salmo salar*) fry culturing system at a fish farm in Chile. Phylogenetic analysis based on 16S rRNA sequences (1394 bp) revealed that strain T-12^T^ belonged to the genus *Flavobacterium*, showing close relationships to *Flavobacterium bernardetii* F-372^T^ (99.48 %) and *Flavobacterium terrigena* DS-20^T^ (98.50 %). The genome size of strain T-12^T^ was 3.28 Mb, with a G+C content of 31.1 mol%. Genome comparisons aligned strain T-12^T^ with *Flavobacterium bernardetii* F-372^T^ (GCA_011305415) and *Flavobacterium terrigena* DSM 17934^T^ (GCA_900108955). The highest digital DNA–DNA hybridization (dDDH) values were 42.6 % with *F. bernardetii* F-372^T^ (GCA_011305415) and 33.9 % with *F. terrigena* DSM 17934^T^ (GCA_900108955). Pairwise average nucleotide identity (ANI) calculations were below the species cutoff, with the best results with *F. bernardetii* F-372^T^ being: ANIb, 90.33 %; ANIm, 91.85 %; and TETRA, 0.997 %. These dDDH and ANI results confirm that strain T-12^T^ represents a new species. The major fatty acids were iso-C_15 : 0_ and C_15 : 1_ω6*с*. Detected polar lipids included phospholipids (*n*=2), aminophospholipid (*n*=1), aminolipid (*n*=1) and unidentified lipids (*n*=2). The predominant respiratory quinone was menaquinone MK7 (80 %) followed by MK-6 (20 %). Phenotypic, chemotaxonomic, and genomic data support the classification of strain T-12^T^ (=CECT 30410^T^=RGM 3222^T^) as representing a novel species of *Flavobacterium*, for which the name *Flavobacterium facile* sp. nov. is proposed.

## Introduction

The genus *Flavobacterium* was proposed a century ago by Bergey *et al*. [1] and is the type genus of the family *Flavobacteriaceae*. This genus comprises 301 species with validly published names listed in the Prokaryotic Names with Standing in Nomenclature (https://lpsn.dsmz.de/genus/flavobacterium; accessed on 18 July 2024).

Members of *Flavobacterium* are Gram-negative, produce yellow-pigmented colonies, are widespread in nature, mainly in water environments, and their various states (glaciers, water, streams, river, etc.), but not in high salinity environments. Species can grow at 4 °C, but their optimal growth temperature is typically between 20–30 °C. They have been isolated from soil and muddy soil samples [2]. Several members are serious pathogens of freshwater fish species, causing significant economic losses in the aquaculture industry worldwide [3,4]. In fish, *Flavobacterium psychrophilum* is the most widely distributed and studied representative of the genus, as it is responsible for the deaths of farmed trout and salmon globally [5].

Chile, being the second largest producer of salmonids after Norway, faces challenges as, according to the 2022 health report from the National Fisheries and Aquaculture Service [6], *F. psychrophilum* is the primary cause of mortality in fish farmed in freshwater. Consequently, monitoring the health status of the fish, the microbiological quality of the water, and the infrastructure of farming centres is crucial to prevent infectious outbreaks. Understanding the bacteria present in aquaculture systems is essential to comprehend the microbiota present at different stages of fish culture. This study reports the isolation and characterization of strain T-12^T^, representing a novel species within the genus *Flavobacterium*, recovered from the cultivation water of a pisciculture system located in the Maule region in 2013.

## Isolation and ecology

In November 2013, water samples were collected from an Atlantic salmon (*Salmo salar*) fry culture system in the Maule region of Chile (35^o^ 25′35.04″ S 71^o^ 39′ 11.5″ W) to analyse the microbiological population present. The samples were analysed using the plate serial dilution technique in an 0.85 % sterile saline solution, with subsequent spreading of 100 µl from each dilution onto tryptone–yeast extract–salts (TYES) agar plates [7]. All microbiological samples were incubated at 18 °C for 8 days, with daily observation for the growth of bacterial colonies. The dominant morphology, represented by strain T-12^T^, was isolated and purified for subsequent species-level identification. Strain T-12^T^ was stored in commercial Criobille tubes (AES Laboratoire) and in cryovial tubes prepared with TYES broth supplemented with 10 % glycerol (v/v), maintained at −80 °C until use.

## 16S rRNA gene phylogeny

For the taxonomic identification of strain T-12^T^, two colonies were picked up from pure culture growth on TYES agar, and genomic DNA was extracted using InstaGene Matrix (Bio-Rad), following the manufacturer's instructions. The 16S rRNA gene was amplified by PCR using the primer pair pA (3′-AGAGTTTGATCCTGGTCAG-5′) and pH (3′-AAGGAGGTGATCCAGCCGCA-5′) [8], and the resulting amplification product was sequenced by Macrogen (Seoul, Republic of Korea). The 16S rRNA sequence was edited in Geneious Prime version 2023.0.4 (https://www.geneious.com), with manual verification in BioEdit version 7.2.5 [9]. Pairwise 16S rRNA gene sequence similarities to the most closely related type strains were determined by Basic Local Alignment Search Tool (blast) analysis against the 16S rRNA gene sequence database in EzBioCloud [10]. Phylogenetic trees were reconstructed for the 16S rRNA sequences of 17 closest type strains of the genus *Flavobacterium* using the neighbour-joining algorithm in mega version 11.011 [11] and Bayesian inference in MrBayes version 3.0B4 [12]. *Imtechella haloterans* K1^T^ was used as an outgroup, according to results obtained by Gavriilidou *et al.* [13]. mega was used to estimate the best-fitting model of nucleotide evolution. The GTR+G+I evolution model was used for Bayesian inference analysis as the best-fitting model according to the Bayesian information criterion [14].

**Fig. 1.**
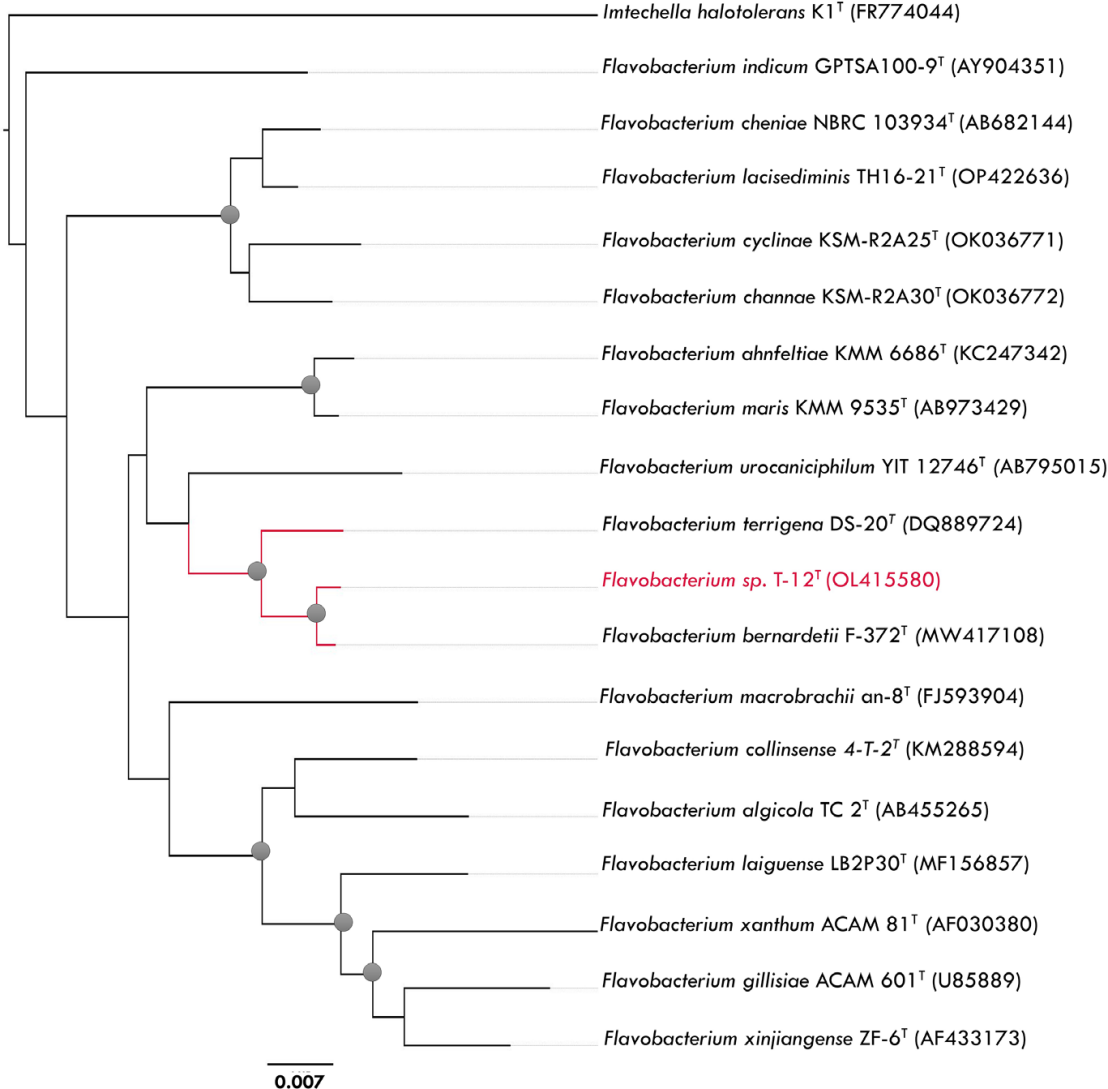
The taxonomic position of *Flavobacterium* sp. T-12^T^ and 17 type strains of the genus *Flavobacterium*. The tree was inferred in mega X with the neighbour-joining algorithm, analysing 1317 bp of the 16S rRNA gene. Evolutionary distances were computed using the *p*-distance, with bootstrap support from 10 000 replicates. Nodes with bootstrap support ≥80 % are highlighted. *Imtechella halotolerans* K1^T^ (FR774044) served as an outgroup.

The 16S rRNA gene sequence of strain T-12^T^ (accession number OL415580) had a length of 1394 bp, and the highest similarity was 99.48 % to *Flavobacterium bernardetii* F-372^T^ (MW417108), followed by 98.50 % to *Flavobacterium terrigena* DS-20^T^ (DQ889724). Phylogenetic analyses based on the 16S rRNA gene clustered strain T-12^T^ within the genus *Flavobacterium.* Both the neighbour-joining and the Bayesian inference phylogenetic algorithms revealed the close phylogenetic relationship of strain T-12^T^ with * F. bernardetii* F-372^T^ in a well-supported clade (bootstrap support, 99 %; posterior probability, 0.99) (Figs 1 and S1, available in the online version of this article.

## Genome analysis, phylogeny and features

The genome sequence of strain T-12^T^ was drafted at the Fraunhofer Chile Research Foundation. Libraries were constructed using the Nextera XT library preparation kit and sequenced on a MiSeq instrument with v3 chemistry and paired-end reads (2×300 cycles) (Illumina). A total 469 471 read pairs were assembled using SPAdes version 3.11.1 [15]. The assembly quality was checked by quast version 5.1 [16]. CheckM version 1.2.2 [17] was used to estimate the completeness (98.49 %) and contamination (1.27 %). The draft genome is 3 281 376 bp, comprising 35 scaffolds (>1000 bp), an N50 value of 287 260 bp, a minimum contig length of 1068 bp, a maximum contig length of 622 914 bp, and a G+C content of 31.1 mol%.

**Fig. 2.**
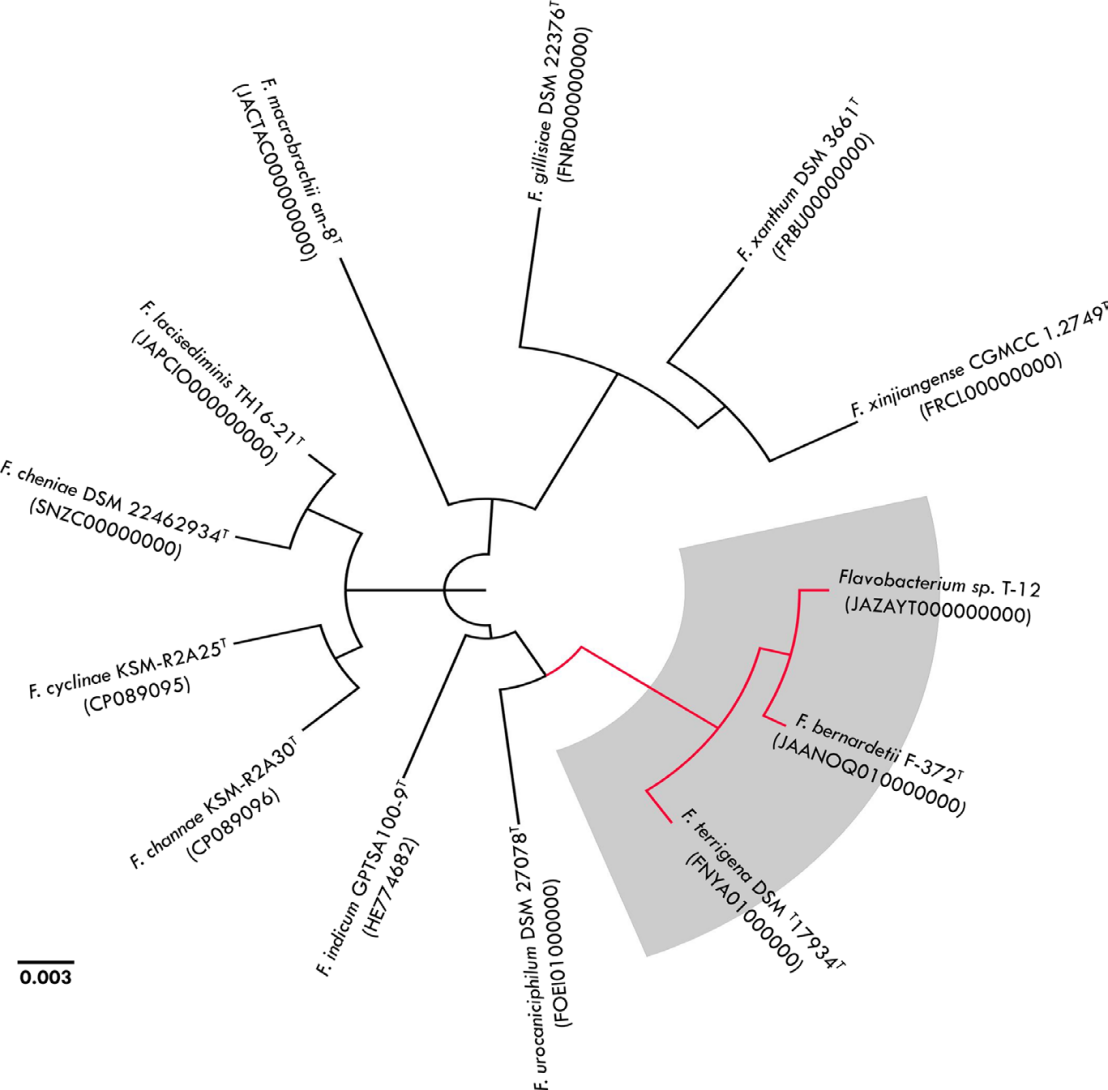
Phylogenetic relationships among *Flavobacterium* sp. T-12^T^ and 12 closely related *Flavobacterium* type strains. The phylogeny was reconstructed using whole-genome sequence data in the Reference Sequence Alignment-Based Phylogeny builder with inference via PhyML. Grey dots signify bootstrap support >80 %.

For taxonomic analysis, the genome sequence data of strain T-12^T^ were uploaded to the Type Strain Genome Server (https://tygs.dsmz.de/user_requests/new) [18]. This analysis identified 12 closely related *Flavobacterium* type strains, which were downloaded from the National Center for Biotechnology Information (https://www.ncbi.nlm.nih.gov/genome) for phylogenetic analysis. The relationships between strain T-12^T^ and the validated 12 type strains with available whole genomes were inferred using realphy via PhyML [19]. The whole-genome phylogenetic tree based on 1162 polymorphic sites was congruent with the 16S rRNA gene tree, showing strain T-12^T^ to be most closely related to *F. bernardetii* F-372^T^ (bootstrap support, 100 %) (Fig. 2).

Pairwise genome comparisons with these 12 *Flavobacterium* type strains showed digital DNA–DNA hybridization (dDDH) values below the recommended cut-off point for species delineation (i.e., 70 %) [20], suggesting that strain T-12^T^ represents a new species. The highest dDDH values were 42.6 % with *F. bernardetii* F-372^T^ (GCA_011305415) and 33.9 % with *F. terrigena* DSM 17934^T^ (GCA_900108955). Pairwise average nucleotide identity (ANI) calculations using the JSpecies Web server [21] were below the species cutoff: < 94 % identity for blast analysis (ANIb), < 96 % MUMer analysis (ANIm), and <0.999 for tetranucleotides frequency analysis (TETRA) [22]. The values obtained for the analyses were below the cutoff range, between 71.44 and 90.33 % for ANIb, between 83.06 and 91.85 % for ANIm, and between 0.821 and 0.997 for TETRA. The best results with *F. bernardetii* F-372^T^ were as follows: ANIb, 90.33 %; ANIm, 91.85 % and TETRA, 0.997 %.

The genome annotation of strain T-12^T^, along with that of its closest related strains, *F. bernardetii* F-372^T^ and *T. terrigena* DSM 17934^T^, was performed by the rast annotation server [23]. The general genomic features of these type strains are shown in Table 1. The richest rast subsystems of these type strains were amino acids and derivatives, cofactors, vitamins, prosthetic groups, pigments, and protein metabolism, showing differences in the number of coding sequences (CDS) included in each subsystem. Genes related to the secretion systems T1SS and T9SS were identified in the genome of strain T-12^T^ using TXSScan [24]. Iron-related protein families were searched in Fegenie software version 1.2 [25], identifying families involved in haemin transport, iron transport, siderophore transport, transcriptional regulation and iron storage. Haemolysins and related proteins containing CBS domains were also found (Table S1). The Resistance Gene Identifier tool (90 % threshold) in the Comprehensive Antibiotic Resistance Database (card) [26] found no resistance genes in strain T-12^T^ .

**Table 1.** General genomic features of the strain T-12^T^ and its closely related *Flavobacterium* species Strains: 1, T-12^T^; 2, *F. bernardetii* F-372^T^ [33]; 3, *F. terrigena* DS-20^T^ [34].

Characteristic	1	2	3
Genome status	Draft	Draft	Draft
Completeness (%)	98.49	98.18	97.86
Contamination	1.27	0.02	0.94
Genome size	3 281 376	3 386 502	3 172 415
G+C content (mol%)	31.1	30.9	31.2
Scaffolds	35	37	18
CDS	3004	3161	2897
rRNA genes	5	4	3
tRNA genes	43	40	39
Subsystems	220	223	218
Richest rast subsystem feature/number of CDS	Amino acids and derivatives (146/808), cofactors, vitamins, prosthetic groups, pigments (130/808), and protein metabolism (94/808)	Amino acids and derivatives (168/869), protein metabolism (130/869), and cofactors, vitamins, prosthetic groups, pigments (124/869)	Amino acids and derivatives (158/831), protein metabolism (132/831), and cofactors, vitamins, prosthetic groups, pigments (122/831)

Virulence factors were searched in the Virulence Factor Database (http://www.mgc.ac.cn/VFs/ [27]) using a 90 % threshold, identifying factors in the following categories antiphagocytosis, iron uptake, secretion system, immune evasion, and stress adaptation (Table S2).

In addition, we performed a pairwise-coding sequence comparison using bidirectional blastp incorporated into the Bacterial and Viral Bioinformatics Resource Center platform (https://www.bv-brc.org/). This comparison used CDS data annotated in strain T-12^T^ (3004 CDS) and coding sequences data annotated in *F. bernardetii* F-372^T^ (3161 CDS). The number of coding sequences conserved in both type strains was 2480, with a percent identity ranging from ~22 to 100 %. The greatest genetic differentiation was observed in proteins in relation to proteins involved in the replication process, carbohydrate metabolic process, siderophore uptake transmembrane transport, and nucleoside biosynthesis. One hundred thirty-four CDS (5.4 %) were implicated in processes such as translation, structural constituent of ribosome, transport and plasma membrane, showing an identity of 100 % (Table S3; https://doi.org/10.6084/m9.figshare.26222963).

## Physiology and chemotaxonomy

For phenotypic and comparative purposes, strain T-12^T^ was grown on various media including Luria–Bertani (Oxoid), nutrient agar (Oxoid), Reasoner's 2A agar (Oxoid), marine agar 2216 (Difco), McConkey agar (Oxoid), thiosulphate–citrate–bile salts–sucrose agar (Oxoid), tryptic soya agar (Difco) and TYES. The agar plates were incubated aerobically at 20±1 °C for 1 week. Abundant growth was observed on TYES agar plates, good growth on marine agar 2216 and tryptic soya agar, weak growth on nutrient agar and Reasoner's 2A agar, and no growth on McConkey agar and thiosulphate–citrate–bile salts–sucrose agar. Colony development on TYES and Reasoner's 2A agar plates presented as orange, circular colonies with entire edges and shiny surfaces (Fig. S2).

**Fig. 3.**
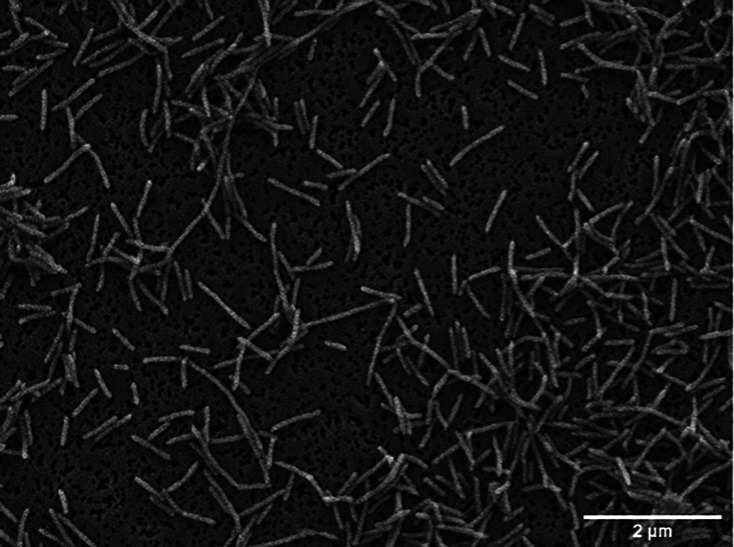
Scanning electron microscopy image of strain T-12^T^ after 48 h of incubation at 20 °C in TYES broth. Bar, 2 µm.

Gram staining and cell morphology were determined using light microscopy at ×1000 magnification with a Motic BA410 Elite microscope. The Gram-negative and rod-shaped cells were 0.6 µm wide and 1.9–5.7 µm long, as observed under a Zeiss Auriga compact field scanning transmission electron microscope (Fig. 3). Gliding motility was not detected in cells cultured for 24 h on Reasoner's 2A plates. Catalase and oxidase activities were tested using 3  % hydrogen peroxide and an oxidase reagent dropper kit (BD BBL), respectively, revealing that strain T-12^T^ was catalase positive but oxidase negative. The presence of flexirubin pigments and Congo red absorption was tested according to Bernardet *et al.* [28], yielding strong positive and negative results, respectively. Optimal growth temperature was defined using Reasoner's 2A plates at different incubation temperatures (i.e., 5, 10, 15, 18, 20, 25, 30 and 37 °C), and growth was observed at 5–25 °C (optimal, 18–20 °C). Tolerance to different pH levels and NaCl concentrations was assessed on Reasoner's 2A agar plates prepared at pH 5–10 (1 pH unit interval; pH was adjusted with 1 N HCl and 1 M NaOH) and with 0–6 % NaCl (w/v), showing best growth at pH 7.0–10.0 (optimal, pH 7.0–9.0) and at 0 % NaCl concentration. Hydrolysis of various substrates was tested on Reasoner's 2A agar plates, including casein (1 % w/v), carboxymethyl cellulose degradation (0.5 % w/v), DNAse (Liofilchem), egg-yolk precipitation (5 % v/v, Liofilchem), gelatin (2 % w/v), l-tyrosine (0.5 % w/v), pectin (0.5 % w/v), starch (0.5 % w/v) and Tweens 20 and 80. There were positive results for gelatin hydrolysis and growth on plates with Tween 80, but no hydrolysis was observed. No growth was noted on the remaining agar plates prepared for hydrolysis tests. Enzymatic activities were assessed using APIZYM, API20E, API20NE and API50CH systems according to the manufacturer's instructions, except that the incubation temperature was set at 20 °C, and the incubation period for the API ZYM assay was 48 h. Alkaline phosphatase, esterase (C4), esterase lipase (C8), leucine and valine arylamidase activities were strongly positive; cystine arylamidase, trypsin and *N*-acetyl-β-glucosaminidase were weakly positive, and lipase (C14), α-chemotrypsin, acid phosphatase, naphthol-AS-BI-phosphohydrolase, α- and β-galactosidase, β-glucuronidase, α-mannosidase, and α-fucosidase were negative. In the API20E and API20NE systems, the only positive test was gelatin hydrolysis, with a weak positive Voges–Proskauer reaction. No reaction was observed in the API50CH galleries even after 1 week of incubation. The main phenotypic characteristics differentiating the Chilean T-12^T^ strain from its closest *Flavobacterium* species are presented in Table 2.

**Table 2.** Differential phenotypic characteristics of strain T-12^T^ and phylogenetically related *Flavobacterium* species Strains: 1, T-12^T^; 2, *F. bernardetii* F-372^T^ [33]; 3, *F. terrigena* DS-20^T^ [34]. +, Positive; −, negative; w, weak positive reaction; nd, not determined.

Characteristic	1	2	3
Country of isolation	Chile	Turkey	Republic of Korea
Year of isolation	2013	2017	2004
Source of isolation	Water system Atlantic salmon fry	Liver diseased rainbow trout	Soil sample
Basal medium	R2A	R2A	NA
Gliding motility	−	−	−
Catalase/oxidase	+/−	+/+	+/+
Flexirubin/Congo red	+/−	+/−	+/−
Temperature range (optimum) for growth (°C)	5–25 (20)	0–25 (25)	10–30 (25)
pH range (optimum) for growth	7.0–10.0 (7.0–9.0)	7.0–9.0 (7.0)	6.0–8.0 (6.5–7.0)
Salt tolerance for growth (%)	0	0–1	nd
Hydrolysis of:			
Casein	−	+	+
DNAse	−	−	−
Starch	−	−	−
Tween 20/Tween 80	−/−	−/+	+/+
Tyrosine	−	nd	+
API ZYM assay:			
Cystine arylamidase	w	+	−
Trypsin	w	w	−
Naphthol-AS-BI-phosphohydrolase	−	+	−
*N*-Acetyl-β-glucosaminidase	w	−	−
Major fatty acids (>10 %)	iso-C_15 : 0_ and C_15 : 1_ω6*с*	iso-C_15 : 0_ and C_15 : 1_ω6*с*	iso-C_15 : 0_, iso-C_17 : 0_ 3-OH and iso-C_17 : 1_ω9*с*
G+C content (mol%)	31.0	30.89	38.2

The fatty acid composition was determined from cells grown on Reasoner's 2A agar plates incubated at 25 °C for 72 h. The analysis was conducted at the Bacterial Section of the Colección Española de Cepas Tipo (CECT, Spain) using the midi Microbial Identification System [29]. The fatty acid content was obtained by GC in an Agilent 6850 system with the Sherlock midi microbial identification system using TSBA6 [30]. The cell compositions of the novel strain and its closest related species are shown in Table 3, and the predominant fatty acids (>10 %) were iso-C_15 : 0_ and C_15 : 1_ω6*с*.

**Table 3.** Cellular fatty acid composition of strain T-12^T^ and the closest *Flavobacterium* species Strains: 1, T-12^T^; 2, *F. bernardetii* F-372^T^ [33]; 3, *F. terrigena* DS-20^T^ [34]. Fatty acids that represent >10% are indicated in bold. tr, Trace; –, not detected.

Fatty acid	1	2	3
**Saturated**			
C_12 : 0_	–	4.5	–
C_13 : 0_	0.8	–	–
C_14 : 0_	–	1.9	–
C_15 : 0_	–	–	4.3
C_16 : 0_	0.8	6.9	–
C_18 : 0_	–	4.9	–
C_18 : 0_ 10-methyl, TBSA	1.5	–	–
C_20 : 0_	–	tr	–
**Unsaturated**			
C_15 : 1_ ω6*с*	**26.8**	**10.0**	1.3
C_17 : 1_ ω6*с*	7.2	3.3	–
C_17 : 1_ ω8*с*	1.6	1.3	–
C_18 : 1_ ω9*с*	–	2.0	–
**Branched-chain**			
iso-C_13 : 0_	–	–	–
iso-C_13 : 1_	1.2	–	–
iso-C_14 : 0_	1.6	2.4	–
iso-C_15 : 0_	**18.3**	**13.2**	**30.1**
iso-C_15 : 1_		4.4	5.2
iso-C_16 : 0_	3.0	7.6	–
iso-C_16 : 1_	–	3.1	2.2
iso-C_16 : 1_ h	1.8	–	–
iso-C_15 : 1_ G	5.4	–	–
iso-C_17 : 1_ ω9с	–	–	11.7
**Hydroxy**			
iso-C_14 : 0_ 3-OH	–	1.4	–
C_15 : 0_ 3-OH	4.0	–	–
iso-C_15 : 0_ 3-OH	6.3	4.1	6.6
iso-C_16 : 0_ 3-OH	2.7	3.6	3.6
C_16 : 0_ 3-OH	1.0	1.6	–
iso-C_17 : 0_ 3-OH	6.1	5.2	16.0
C_17 : 0_ 3-OH	1.5	–	–
anteiso-C_15 : 0_	1.0	1.2	–
Summed features*			
3	4.0	–	2.4
9	4.0	–	–

†*, Summed features 33 comprised C_16 : 1_ ω6*c* and/or C_16 : 1_ ω7*c* for strain T-12T and iso-C_16 : 1_ ω7с and/or iso-C_15 : 0_ 2-OH for *F. terrigena* DS-20T; Ssummed feature 99 comprised iso-C_17 : 1_ω9*с* and/or C_16 : 0_ 10-methyl.

Respiratory menaquinone and polar lipid determinations were performed using cells grown in TYES broth at 20 °C for 48 h. The cells, harvested by centrifugation and resuspended in an isopropanol:water solution (1 : 1) were analysed at DSMZ Services, Leibniz – Institute DSMZ (Deustcshe Sammlung von Mikroorganismen und Zellkulturn GmbH, Braunschweig, Germany) following standard methods [31,32]. The menaquinones detected as respiratory quinones in strain T-12^T^ were predominantly MK-7 (80 %) and MK-6 (20 %). The identified polar lipids consisted of two phospholipids, two unidentified lipids, one aminophospholipid and one aminolipid (Fig. S3).

Based on the results of the polyphasic taxonomic analysis and the distinctions identified in comparison with closely related species, strain T-12^T^ (=CECT 30410^T^=RGM 3222^T^) is proposed as a representative of a new bacterial species, *Flavobacterium facile* sp. nov.

## Description of *Flavobacterium facile* sp. nov.

*Flavobacterium facile* (fa’ci.le. L. neut. adj. *facile*, ready, quick, referring to the ease of cultivation of the organism).

Colony development on Reasoner's 2A agar plates is orange, circular, with entire edges and shiny surface, and diameters ranging from 1 to 2 mm. Cells are Gram-negative rods, measuring 0.6 µm wide and 1.9–5.7 µm long after 48 h of incubation at 20 °C. Cells are non-motile, flexirubin positive, and Congo red negative. Catalase is positive, while oxidase is negative. Growth was observed on TYES agar, marine agar 2216, tryptic soya agar, nutrient agar, and Reasoner's 2A agar. The type strain grew at 5–25 °C (optimal, 18–20 °C), on Reasoner's 2A with no NaCl added, and at pH 7.0–10.0 (optimal, pH 7.0–9.0). Hydrolysis of gelatin is observed, but not of casein, DNAse, Tween 20 or 80, starch nor l-tyrosine. No growth on Reasoner's 2A agar plates supplemented with carboxymethyl cellulose degradation, pectin, or egg yolk. Strong enzymatic activity is observed with alkaline phosphatase, esterase (C4), esterase lipase (C8), leucine and valine arylamidase; cystine arylamidase, trypsin and *N*-acetyl-β-glucosaminidase are weakly positive; and lipase (C14), α-chemotrypsin, acid phosphatase, naphthol-AS-BI-phosphohydrolase, α- and β-galactosidase, β-glucuronidase, α-mannosidase, and α-fucosidase are negative. In the API20E and API20NE system, the only positive test is gelatin hydrolysis, with a weak positive Voges–Proskauer reaction. No reactions are obtained in the API50CH galleries, even after 1 week of incubation. The main fatty acids (>10 %) are iso-C_15 : 0_ and C_15 : 1_ ω6*c*. The predominant respiratory quinones are MK-7 (80 %) and MK-6 (20 %). The genomic G+C content is 31.1 mol%.

The type strain, T-12^T^ (=CECT 30410^T^=RGM 3222^T^), was isolated from water samples collected from farm-cultured Atlantic salmon fry in Chile.

The GenBank accession number for the 16S rRNA gene sequence of strain T-12^T^ is OL415580 and the genome assembly has the accession number JAZAYT000000000.1.

## supplementary material

10.1099/ijsem.0.006468 Uncited Supplementary Material 1.
