# Supplementary material for: Flavobacterium facile sp. nov., isolated from water system of Atlantic salmon (Salmo salar) fry cultured in Chile
Source: Int J Syst Evol Microbiol. 2024 Jul 26;74(7):006468. doi: 10.1099/ijsem.0.006468 (PMC11281480; doi:10.1099/ijsem.0.006468)
Supplement: Uncited Supplementary Material 1. [file ijsem-74-06468-s001.pdf]

Flavobacterium facile sp. nov., isolated from water system of Atlantic salmon (*Salmo salar*) fry cultured in Chile

1.1. Authors name

Rute Irgang[1,2], Mónica Saldarriaga-Córdoba[2,3], Matías Poblete-Morales[1], Ruben Avendaño-Herrera[1,2,4]*,

1.2. Affiliations

[1]Universidad Andrés Bello, Laboratorio de Patología de Organismos Acuáticos y Biotecnología Acuícola, Facultad de Ciencias de la Vida, Viña del Mar, Chile.

[2]Interdisciplinary Center for Aquaculture Research (INCAR), Viña del Mar, Chile.

[4] Centro de Investigación en Recursos Naturales y Sustentabilidad, Universidad Bernardo O'Higgins, Santiago, Chile.

[4]Centro de Investigación Marina Quintay (CIMARQ), Universidad Andrés Bello, Quintay, Valparaíso, Chile.

1.3. Corresponding author

R. Avendaño-Herrera. Universidad Andrés Bello, Quillota 980, Piso 4-Torre C, Viña del Mar, Chile. Tel.: +56 968989187; E-mail: reavendano@yahoo.com or ravendano@unab.cl

Supplementary Figure

Figure S1 The taxonomic position of *Flavobacterium facile* sp. T-12^{T} among the 18 type species of the genus *Flavobacterium*. Inference was conducted in the MrBayes 3.2.7 program using the Bayesian inference algorithm, analyzing 1,317 bp of the 16S rRNA gene. The evolutionary model employed was GTR+G+I. Nodes with a posterior probability ≥0.95 are highlighted. *Imtechella halotolerans* K1^{T} (FR774044) was used as an external group.

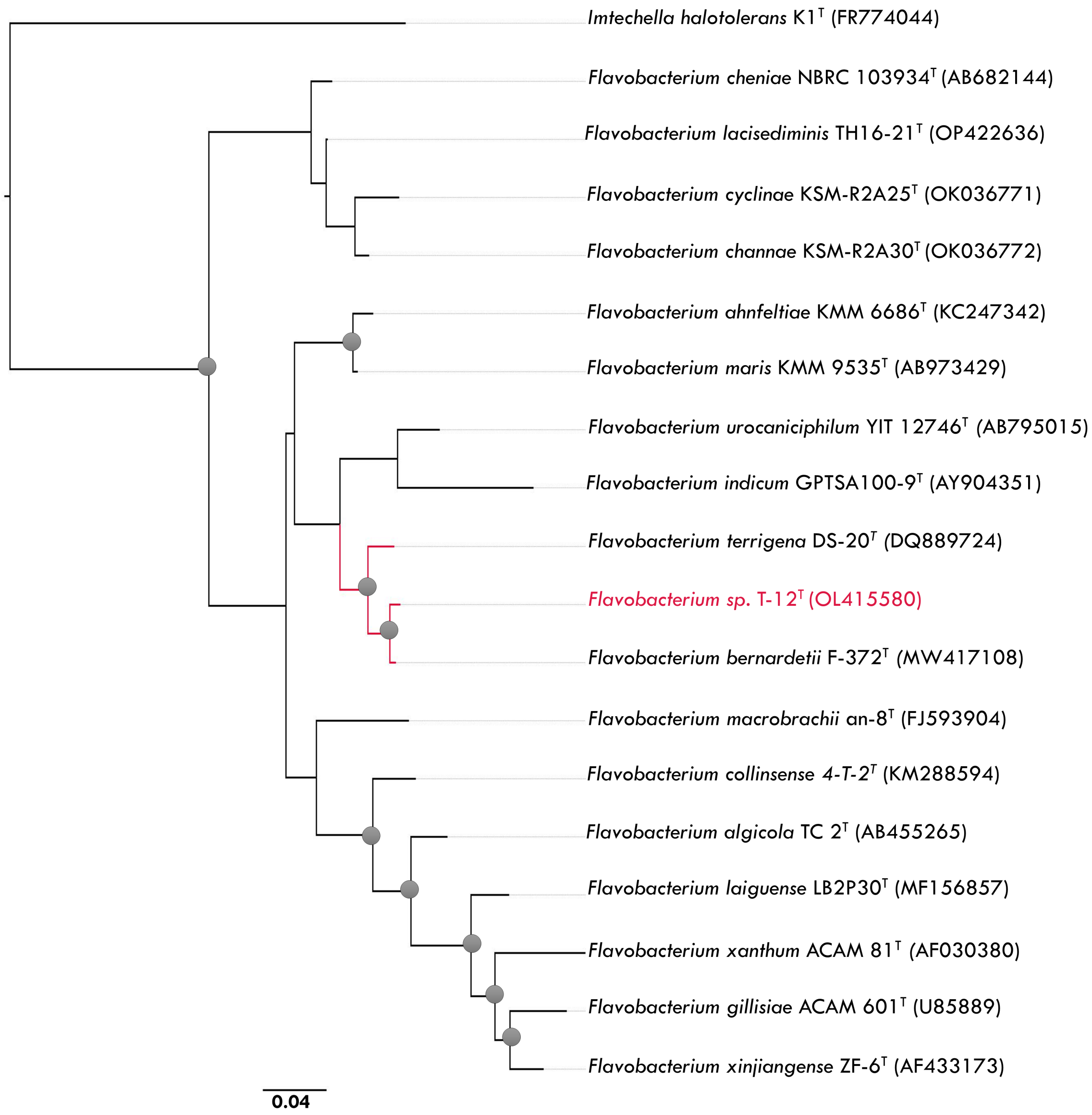

Figure S2 Colonies grown on TYES (left) and R2A (right) agar plates incubated at 20°C for 48 h.

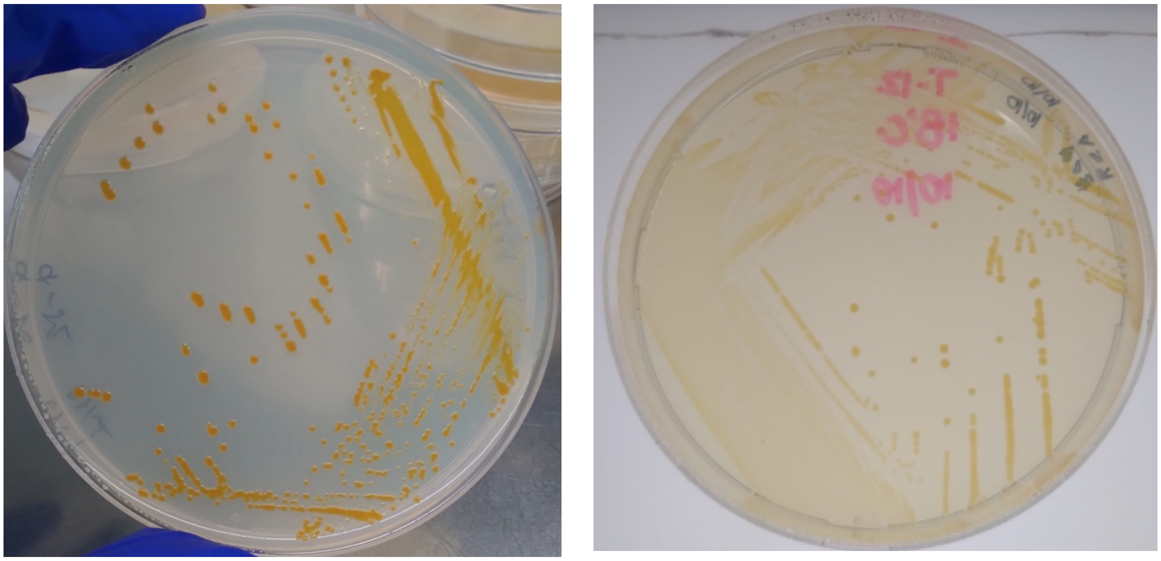

Figure S3 Polar lipids profile of *Flavobacterium facile* T-12^T separated by two-dimensional silica gel thin layer chromatography. Total lipid material was detected using molybdatophosphoric acid, and specific functional groups were identified using spray reagents specific for defined functional groups. APL, aminophospholipid; AL, aminolipid; PL, phospholipid; L, unidentified lipids.

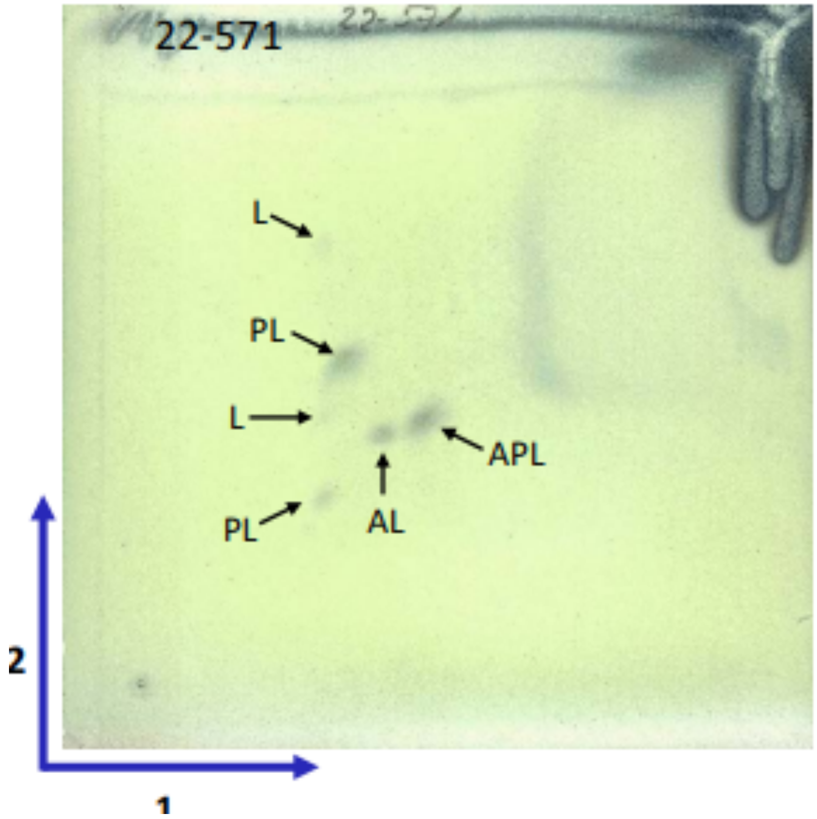

Table S1. Summary of CDS sequences related with secretion system and iron related protein families

Type	Function	Hit	Accession number
Secretion system T1SS	ATP-binding cassette domain-containing protein	*F. bernardetii*	WP_166125712
Secretion system T1SS	HlyD family efflux transporter periplasmic adaptor subunit	*F. bernardetii*	WP_166125711
Secretion system T1SS	TolC family protein	*F. bernardetii*	WP_166124845
Secretion system T1SS	peptidase domain-containing ABC transporter	*F. bernardetii*	P_166129347
Secretion system T1SS	Outer membrane protein TolC	*F. bernardetii*	WP_166124845
Secretion system T9SS	Gliding motility lipoprotein GldK	*F. bernardetii*	WP_166130435
Secretion system T9SS	Gliding motility-associated protein GldL	*F. bernardetii*	WP_166130439
Secretion system T9SS	Gliding motility-associated protein GldM	*F. bernardetii*	WP_166130443
Secretion system T9SS	Gliding motility associated protien GldN	*F. bernardetii*	WP_166130448
Secretion system T9SS	Gliding motility lipoprotein GldJ	*F. bernardetii*	WP_166124497
Secretion system T9SS	Gliding protein	*F. bernardetii*	WP_225875176
Secretion system T9SS	Cell surface protein SprA	*F. difficile*	WP_235922487
Secretion system T9SS	Porin family protein	*F. bernardetii*	WP_166130723
Secretion system T9SS	Type IX secretion system outer membrane channel protein PorV	*F. bernardetii*	WP_166124501
Secretion system T9SS	Type IX secretion system sortase PorU	*F. bernardetii*	WP_166124499
Secretion system T9SS	Type IX secretion system protein PorQ	*F. difficile*	WP_166076656
hemin transport	Hemophore HmuY	*F. bernardetii*	WP_166129130

iron transport	ZIP family metal transporter	*F. bernardetii*	WP_166129111
iron transport	Ferrous iron transport protein B	*F. bernardetii*	WP_166129095
iron transport	Ferrous iron transport protein A	*F. urocaniciphilum*	WP_177177180
siderophore transport	MotA/TolQ/ExbB proton channel family protein	*F. bernardetii*	WP_187003034
siderophore transport	Biopolymer transport protein ExbD	*F. difficile*	WP_166078117
siderophore transport	ABC transporter ATP-binding protein	*F. bernardetii*	WP_166124715
siderophore transport	ABC transporter substrate-binding protein	*F. bernardetii*	WP_166124718
siderophore transport	Iron ABC transporter permease	*F. terrigena*	WP_091308035
siderophore transport	TonB-dependent receptor	*F. bernardetii*	WP_166129126
siderophore transport	Energy transducer TonB	*F. difficile*	WP_166077035
transcriptional regulation	Ferric uptake regulator Fur	*F. difficile*	WP_166076410
transcriptional regulation	AraC family transcriptional regulator	*F. difficile*	WP_166076445
transcriptional regulation	Sigma-70 family RNA polymerase sigma factor	*F. bernardetii*	WP_166128437
transcriptional regulation	Metal-dependent transcriptional regulator	*F. bernardetii*	WP_166129114
iron storage	Ferritin like diiron-binding domain	*F. terrigena*	WP_091308735
iron storage	DNA starvation/stationary phase protection protein	*F. terrigena*	WP_091308735
	Hemolysins proteins containing CBS domains	*F. bernardetii*	WP_166130469

Table S2. Virulence-associated features of strain T-12^T using the VFDB database.

VF class	Virulence factor	Strand	Rast annotation	E-value	% Identity	% Coverage	Description
Adherence	GroEL	-	Heat shock protein 60 kDa family chaperone GroEL	0	100.00	100.00	WP_166130397, chaperonin GroEL [*Flavobacterium bernardetii*]
Iron uptake	Heme biosynthesis	+	hemB	0	99.39	100.00	WP_166129411, porphobilinogen synthase [*Flavobacterium bernardetii*]
Iron uptake	Heme biosynthesis	-	hemL	0	97.43	100.00	WP_166127051, glutamate-1-semialdehyde 2,1-aminomutase [*Flavobacterium bernardetii*]
Secretion system	T4SS effectors	-	Enoyl-[acyl-carrier-protein] reductase [NADH]	0	95.44	99.00	WP_091312575, trans-2-enoyl-CoA reductase family protein [*Flavobacterium terrigena*]
Secretion system	T6SS-II	-	Chaperone protein ClpB (ATP-dependent unfoldase)	0	99.07	100	WP_166124899, ATP-dependent chaperone ClpB [*Flavobacterium bernardetii*]
Adherence and invasion	EF-Tu	-	Translation elongation factor Tu	0	100	100.00	WP_166127174, elongation factor Tu [*Flavobacterium bernardetii*]
Antiphagocytosis	Capsular polysaccharide rmlC	-	dTDP-4-dehydrorhamnose 3,5-epimerase	9.00E-126	93.99	95.00	WP_166077868, dTDP-4-dehydrorhamnose 3,5-epimerase [*Flavobacterium difficile*]

Colonization and Immune evasion	Capsule biosynthesis and transport	-	UDP-glucuronate decarboxylase	0	98.78	100.00	WP_166128527, UDP-glucuronic acid decarboxylase family protein [*Flavobacterium bernardetii*]
Enzyme	Streptococcal enolase	+	Enolase	0	99.07	100	WP_091314265, phosphopyruvate hydratase [*Flavobacterium terrigena*]
Glycosylation system	N-linked protein glycosylation	+	Lipid carrier: UDP-N-acetylgalactosaminyltransferase	5.00E-138	95.02	100	WP_166124567, sugar transferase [Flavobacterium bernardetii]
Immune evasion	Polysaccharide capsule	+	UDP-glucose 4-epimerase	0	94.85	99	WP_166124545, SDR family oxidoreductase [*Flavobacterium bernardetii*]
Immune evasion	Polysaccharide capsule	+	UDP-N-acetyl-D-glucosamine 6-dehydrogenase	0.00E+00	96.7	96	WP_177169108, nucleotide sugar dehydrogenase [*Flavobacterium terrigena*]
Immune evasion	Polysaccharide capsule	+	UDP-N-acetylglucosamine 4,6-dehydratase	0.00E+00	98.27	100	WP_166124563, polysaccharide biosynthesis protein [*Flavobacterium bernardetii*]
Immune evasion	Capsule	-	Glucose-1-phosphate thymidylyltransferase	0.00E+00	98.6	100	WP_091311223, glucose-1-phosphate thymidylyltransferase RfbA [*Flavobacterium terrigena*]
Stress adaptation	Catalase-peroxidase	+	Catalase-peroxidase KatG	0	96.72	100	WP_166126283, catalase/peroxidase HPI [*Flavobacterium bernardetii*]
